# Cecal Perforation Presenting as Apparent Peritoneal Carcinomatosis: Distinguishing Acute Peritonitis From Malignant Mimicry

**DOI:** 10.7759/cureus.105495

**Published:** 2026-03-19

**Authors:** Newton Rahming, Norah Daghestani, Shinelle De Almeida, Imad N Farhat, Mhadhumithaa Naresh, Frederick Tiesenga

**Affiliations:** 1 Surgery, Caribbean Medical University, Willemstad, CUW; 2 Medicine, St. George's University School of Medicine, St. George’s, GRD; 3 Emergency Medicine, All Saints University, Roseau, DMA; 4 Surgery, St. George's University School of Medicine, Chicago, USA; 5 Medicine, St. George's University School of Medicine, Chicago, USA; 6 General Surgery, West Suburban Medical Center, Chicago, USA

**Keywords:** bowel perforation, carcinomatosis, cecal perforation, exploratory laparotomy, postoperative complication

## Abstract

Cecal perforation is a rare but life-threatening surgical emergency that may present with radiologic and intraoperative findings highly suggestive of peritoneal carcinomatosis. This overlap may create substantial diagnostic uncertainty, particularly in patients with systemic symptoms such as weight loss, anemia, and ascites. We report the case of a 39-year-old male patient who presented with sepsis, abdominal pain, and computed tomography (CT) findings concerning for carcinomatosis, including ascites and mesenteric nodularity. Exploratory laparotomy revealed diffuse fibrinous exudates and peritoneal studding highly suspicious for malignant dissemination. However, histopathologic evaluation of peritoneal, mesenteric, omental, and cecectomy specimens demonstrated only acute and chronic inflammation, focal fat necrosis, abscess formation, granulation tissue, and reactive mesothelial changes, with no evidence of dysplasia or malignancy. This case highlights the diagnostic challenge posed by inflammatory mimics of carcinomatosis and underscores the essential role of histopathology in guiding management in complex abdominal emergencies.

## Introduction

Cecal perforation is a rare but severe surgical emergency associated with diffuse peritonitis, sepsis, and substantial morbidity. Mortality rates for colonic perforation range from 6% to 33%, depending on the severity of contamination, comorbidities, and timing of intervention [1,2]. The clinical presentation may be non-specific, and radiologic findings often overlap with other intra-abdominal pathologies, including malignancy. This diagnostic ambiguity is particularly pronounced when peritoneal nodularity and ascites are present, as these features are frequently associated with peritoneal carcinomatosis.

Peritoneal carcinomatosis represents metastatic dissemination along peritoneal surfaces and is most commonly linked to advanced gastrointestinal or gynecologic malignancies [3,4]. Computed tomography (CT) findings including ascites, peritoneal thickening, nodularity, and omental caking are traditionally considered hallmarks of carcinomatosis; however, these features lack specificity. Peritoneal carcinomatosis refers to the spread of malignant tumor cells across the lining of the abdominal cavity, often producing nodular deposits and ascites. In contrast, acute peritonitis represents inflammation of the peritoneal surfaces, typically caused by infection or perforation of an abdominal organ. Although these conditions arise from fundamentally different mechanisms, their imaging and intraoperative appearances can overlap significantly.

Several non-neoplastic conditions including bacterial peritonitis, perforated viscus, granulomatous disease, and reactive mesothelial hyperplasia may closely mimic carcinomatosis radiographically and intraoperatively [5,6]. As a result, distinguishing malignant from inflammatory peritoneal disease can be challenging, particularly in emergent surgical settings where rapid decision-making is required.

This case illustrates the diagnostic complexity inherent in differentiating acute peritonitis from peritoneal carcinomatosis. Despite clinical, radiologic, and intraoperative findings strongly suggestive of malignancy, histopathology ultimately revealed a benign inflammatory process secondary to cecal perforation. The case highlights the importance of maintaining a broad differential diagnosis and relying on tissue confirmation to guide management.

## Case presentation

A 39-year-old Spanish-speaking man with no known medical history presented with one day of worsening abdominal pain. The pain began in the right lower quadrant and progressed to diffuse, severe discomfort. Associated symptoms included subjective fever, nausea, one episode of non-bloody emesis, diarrhea earlier in the week, and unintentional weight loss over several months, which he “couldn’t quantify” according to the hospitalist note. Although the exact amount of weight loss could not be quantified, the patient was reportedly functioning independently prior to this acute illness and was able to ambulate and perform activities of daily living without assistance. He also reported decreased appetite and drenching night sweats. Social history was notable for daily alcohol use and intermittent intranasal cocaine use.

On arrival, he was febrile (100.3°F) and tachycardic (heart rate (HR) 128). Examination revealed diffuse abdominal tenderness with rebound. The patient was triaged as an Emergency Severity Index (ESI) level 2 due to tachycardia, fever, and concern for intra-abdominal sepsis. Initial management included aggressive intravenous fluid resuscitation, broad-spectrum intravenous antibiotics (piperacillin-tazobactam), blood transfusion for severe anemia, and urgent surgical consultation. Laboratory evaluation (Table 1) demonstrated leukocytosis (20 × 10⁹/L), severe microcytic anemia (hemoglobin (Hgb) 6.9 g/dL), thrombocytosis (605 × 10⁹/L), hypoalbuminemia (2.2 g/dL), elevated procalcitonin (44.6 ng/mL), and an international normalized ratio (INR) of 1.5. Cancer antigen 19-9 (CA 19-9) was mildly elevated at 53 U/mL, while carcinoembryonic antigen (CEA) and alpha-fetoprotein (AFP) were normal.

**Table 1:** Laboratory evaluation Leukocytosis, severe microcytic anemia, thrombocytosis, hypoalbuminemia, elevated procalcitonin, INR 1.5, and CA 19-9 53 U/mL, while CEA and AFP were normal. INR: international normalized ratio; CEA: carcinoembryonic antigen; AFP: alpha-fetoprotein; CA 19-9: cancer antigen 19-9

Test	Result	Reference range
White blood cell count	20.0 × 10⁹/L	4.5-11.0 × 10⁹/L
Hemoglobin	6.9 g/dL	13-17 g/dL
Platelets	605 × 10⁹/L	150-400 × 10⁹/L
Albumin	2.2 g/dL	3.5-5.5 g/dL
Procalcitonin	44.6 ng/mL	<0.1 ng/mL
INR	1.5	0.9-1.1
CA 19-9	53 U/mL	<37 U/mL

**Figure 1:**
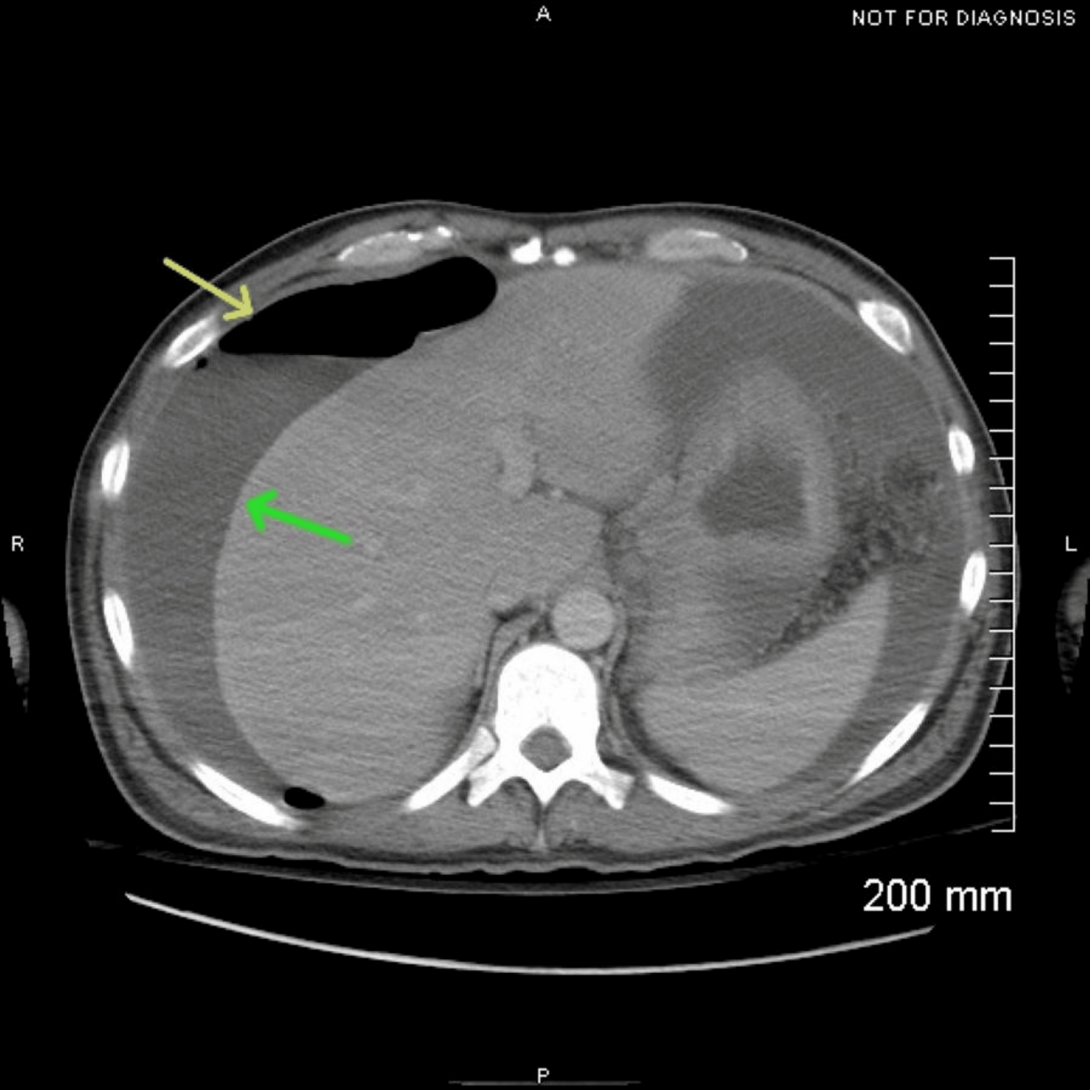
Perihepatic pneumoperitoneum with ascites on CT Axial contrast-enhanced CT demonstrating pneumoperitoneum (yellow arrow) with adjacent perihepatic ascites (green arrow). CT: computed tomography

**Figure 2:**
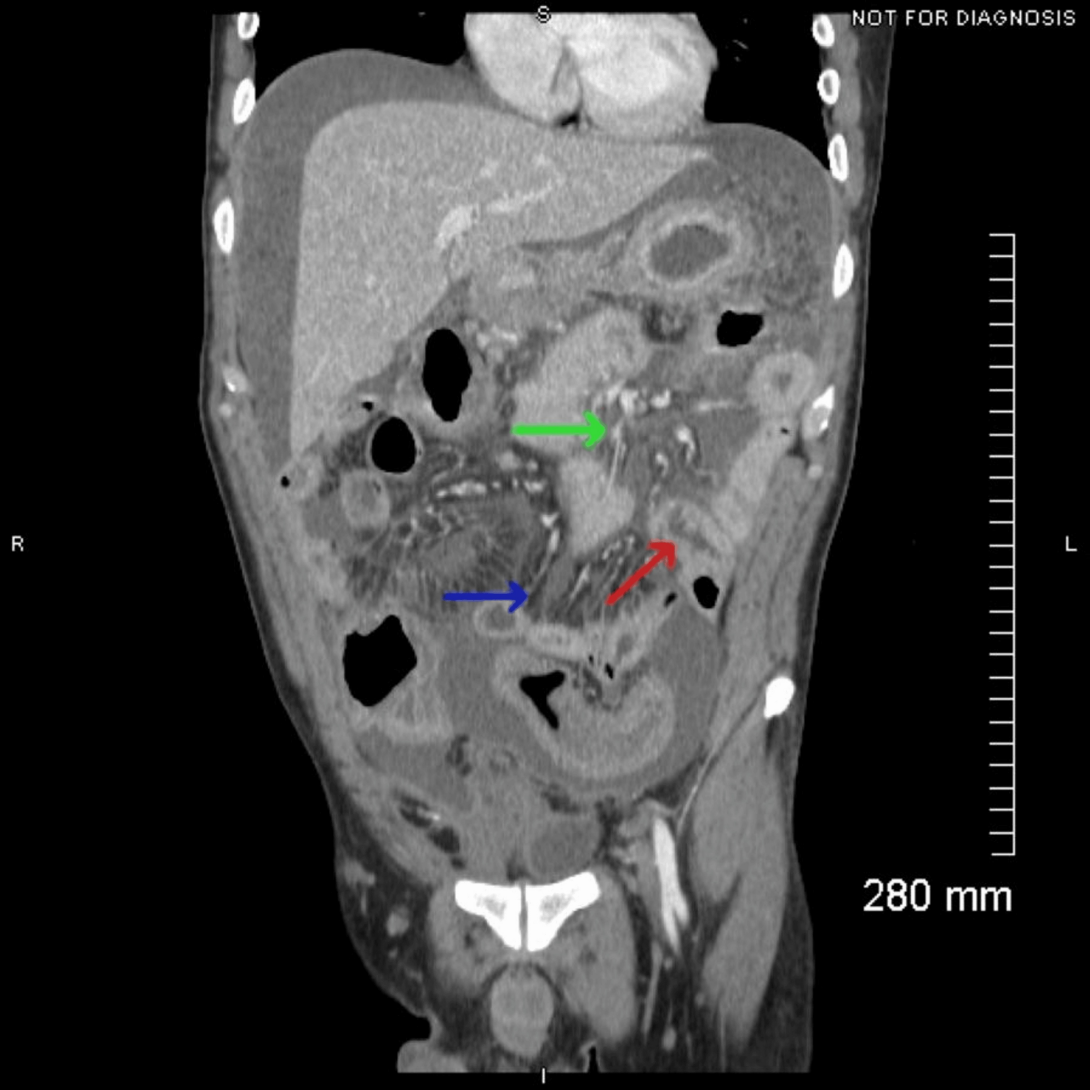
Bowel wall thickening with mesenteric nodularity and inflammatory change Coronal contrast-enhanced CT of the abdomen and pelvis demonstrating segmental bowel wall thickening (red arrow), mesenteric nodularity (green arrow), and surrounding inflammatory fat stranding (blue arrow). CT: computed tomography

Contrast-enhanced CT of the abdomen and pelvis demonstrated an intraperitoneal free air layering anterior to the liver with associated perihepatic ascites (Figure 1), findings concerning for bowel perforation in the appropriate clinical context. Additional evaluation shown in Figure 2 revealed segmental bowel wall thickening involving multiple small-bowel loops with adjacent mesenteric nodularity and prominent inflammatory fat stranding. While these findings may be seen in an inflammatory or infectious process, the presence of mesenteric nodularity also raised concern for peritoneal carcinomatosis. Further evaluation with operative exploration and histopathologic analysis was required for definitive diagnosis.

**Figure 3:**
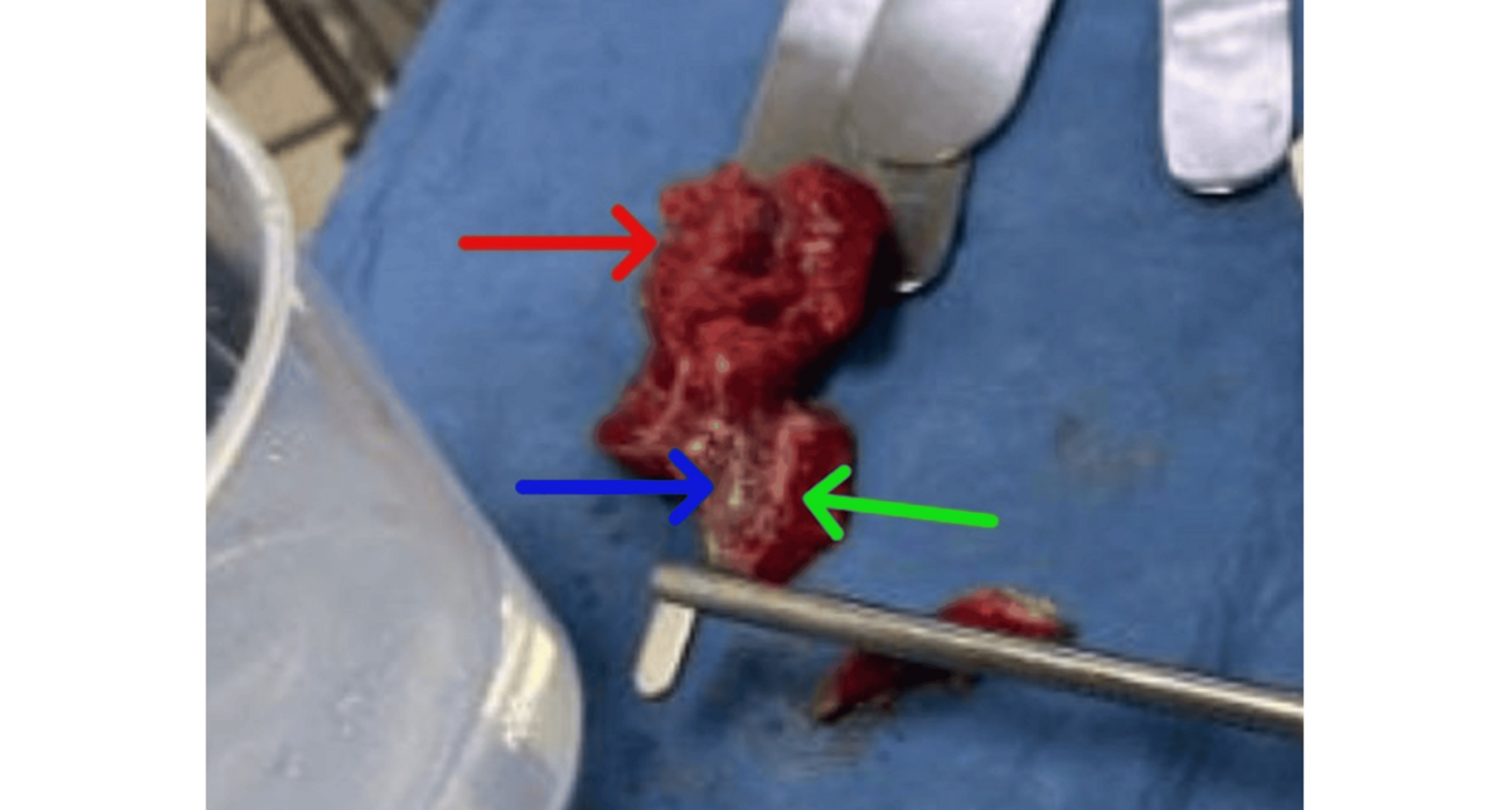
Intraoperative nodular lesions of the omentum and mesentery Intraoperative photograph of excised omental and mesenteric tissue demonstrating irregular nodular lesions (red arrow), areas of hemorrhagic or necrotic change (blue arrow), and surrounding inflamed fibrofatty tissue (green arrow), grossly mimicking peritoneal carcinomatosis.

Given radiographic free air and clinical deterioration, the patient underwent emergent exploratory laparotomy. Upon entering the peritoneal cavity, the surgeon encountered approximately 3 L of contaminated ascitic fluid and diffuse fibrinous exudates. The operative report described “random peritoneal masses” and “studding in the omentum and mesentery,” findings that closely resembled carcinomatosis (Figure 3).

The stomach, duodenum, and colon appeared markedly thickened and foreshortened. A focal cecal perforation was identified as the source of contamination. Representative peritoneal and mesenteric lesions were excised, a partial omentectomy was performed, the perforation was closed with a stapling device, and a diverting loop ileostomy was created.

**Figure 4:**
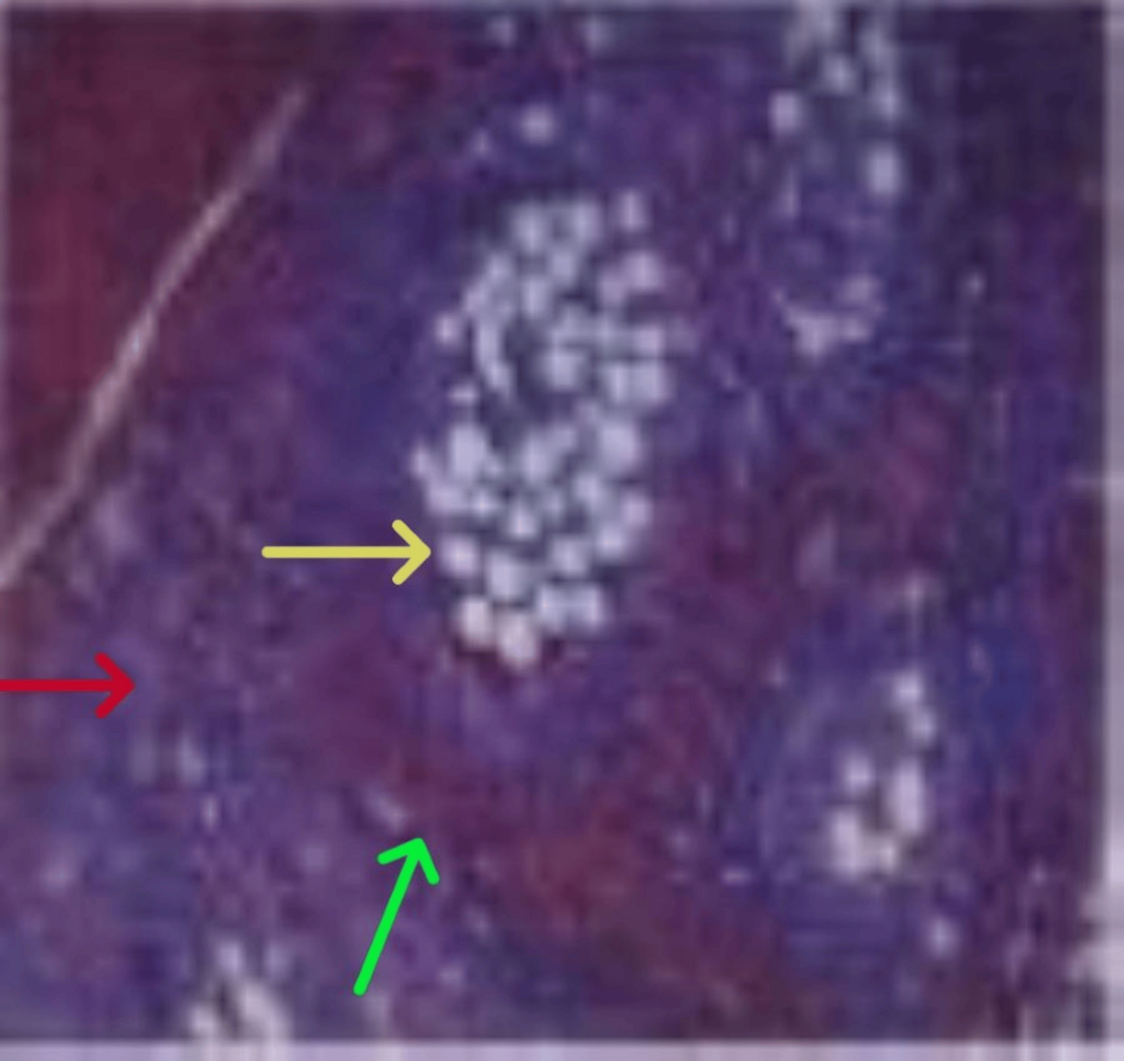
Fat necrosis and inflammatory change on histopathology Histologic section demonstrating fibroadipose tissue with fat necrosis (yellow arrow), associated inflammatory infiltrates (red arrow), and reactive stromal changes (green arrow). No evidence of dysplasia or malignancy was identified.

Microscopic evaluation of the excised peritoneal and mesenteric lesions demonstrated inflammatory and necrotic changes corresponding to the grossly tumor-like implants identified intraoperatively (Figure 4).

Postoperatively, the patient improved with broad-spectrum antibiotics, blood transfusions, and supportive care. Infectious disease, oncology, and gastroenterology were consulted. Fungal and mycobacterial studies were negative. His clinical status gradually stabilized over the next few days. The patient was subsequently discharged in stable condition with outpatient surgical follow-up. At follow-up evaluation several weeks later, he remained clinically stable with no evidence of malignancy on pathology review or imaging. The sequence of clinical evaluation, operative management, and recovery is summarized in Table 2.

**Table 2:** Clinical timeline Clinical timeline of patient presentation, diagnosis, and management. CT: computed tomography

Timepoint	Event
Day 0	Emergency department presentation with abdominal pain and sepsis
Hour 1	Laboratory evaluation revealing leukocytosis and severe anemia
Hour 2	CT abdomen/pelvis showing pneumoperitoneum and ascites
Hour 6	Emergent exploratory laparotomy identifying cecal perforation
Postoperative days 1-3	Stabilization with antibiotics and supportive care
Follow-up	Patient clinically stable with no malignancy identified

## Discussion

Differentiating acute peritonitis from peritoneal carcinomatosis can be challenging because both conditions share overlapping clinical, radiologic, and intraoperative features. This diagnostic ambiguity was central to the clinical dilemma in the present case. The patient exhibited several findings commonly associated with carcinomatosis, including unintentional weight loss, night sweats, anemia, thrombocytosis, ascites, and mesenteric nodularity. These systemic and radiologic features are frequently reported in malignant peritoneal disease, particularly in gastrointestinal and gynecologic cancers [3-6]. The mild elevation in CA 19-9 (53 U/mL) further contributed to the concern for malignancy. However, in gastrointestinal malignancies such as pancreatic or advanced peritoneal cancer, CA 19-9 levels frequently rise into the hundreds or thousands of units per milliliter. Mild elevations such as those observed in this case are non-specific and are commonly seen in inflammatory conditions including peritonitis, biliary disease, and other benign gastrointestinal processes [7].

Radiologically, the patient’s CT demonstrated large-volume ascites, bowel wall thickening, and mesenteric nodularity findings that are classically associated with peritoneal carcinomatosis. Cho and Kim emphasize that peritoneal carcinomatosis typically manifests on CT as “peritoneal nodularity, thickening, and ascites,” but they also note that these findings are not specific and may be seen in inflammatory conditions such as peritonitis or granulomatous disease [5]. This overlap is particularly problematic in the setting of bowel perforation, where reactive mesothelial changes and inflammatory exudates can mimic metastatic implants.

Intraoperative findings further heightened suspicion for carcinomatosis. The operative report described “random peritoneal masses” and “studding in the omentum and mesentery,” along with a diffusely thickened and foreshortened colon. These gross findings are strikingly similar to the appearance of metastatic peritoneal disease, in which tumor implants create nodular, plaque-like deposits across peritoneal surfaces. Several non-neoplastic inflammatory processes, including bacterial peritonitis and fat necrosis, may produce peritoneal thickening, nodularity, and ascites that closely resemble peritoneal carcinomatosis on imaging and intraoperatively [5,6]. In this case, the surgeon’s description of “a large amount of fibrinous fluid and contaminated appearing fluid” and “studding in the omentum and mesentery” closely paralleled the operative appearance of carcinomatosis.

Despite these malignancy-suggestive features, several key findings ultimately distinguished acute peritonitis from true peritoneal carcinomatosis. Foremost among these was histopathology. All specimens demonstrated acute and chronic inflammation, focal fat necrosis, abscess formation, granulation tissue, and reactive mesothelial changes, with no evidence of dysplasia or malignancy. The pathology report explicitly stated: “Negative for malignancy” and “Negative for dysplasia or malignancy.” These findings are characteristic of severe inflammatory peritonitis rather than metastatic disease. Reactive mesothelial hyperplasia, in particular, is a well-documented mimic of carcinomatosis and can produce nodularity that resembles tumor implants both radiographically and intraoperatively [8]. The underlying cause of the cecal perforation in this patient was not definitively identified. Histopathologic evaluation demonstrated acute and chronic inflammation, fat necrosis, and abscess formation without evidence of malignancy, inflammatory bowel disease, or granulomatous infection. In the absence of these findings, the perforation was most consistent with an acute inflammatory or infectious process leading to focal weakening of the bowel wall and secondary rupture. Similar mechanisms have been described in cases of severe localized peritonitis or transient ischemic injury to the bowel [2,9].

Additionally, no primary tumor was identified. The colon was markedly inflamed and thickened but showed no evidence of carcinoma. Tumor markers such as CEA and AFP were within normal limits, and the patient’s clinical improvement with broad-spectrum antibiotics and surgical source control further supported an inflammatory rather than malignant etiology. Peritoneal carcinomatosis does not resolve with antimicrobial therapy, whereas acute peritonitis typically improves once the source of contamination is addressed [9].

Histopathology demonstrated acute and chronic inflammation without evidence of malignancy, granulomatous disease, or inflammatory bowel disease. No findings suggestive of Crohn’s disease, stercoral perforation, or neoplastic obstruction were identified. In the absence of these conditions, the perforation was most consistent with an acute inflammatory or infectious process resulting in focal weakening of the bowel wall and secondary rupture. Similar mechanisms have been described in cases of severe localized peritonitis or transient ischemic injury to the bowel [9].

This case highlights the limitations of relying solely on imaging and intraoperative appearance when evaluating suspected peritoneal malignancy. Ascites, peritoneal nodularity, and systemic symptoms such as weight loss may strongly suggest carcinomatosis but can also occur in severe inflammatory states such as bowel perforation with diffuse peritonitis. In this patient, the combination of pneumoperitoneum, contaminated ascites, and inflammatory nodularity ultimately reflected a benign inflammatory process rather than metastatic disease. Histopathologic evaluation remains the definitive diagnostic modality when imaging and operative findings are ambiguous. Recognition of inflammatory mimics is critical to avoid premature oncologic conclusions and to ensure that management remains focused on prompt surgical source control and stabilization.

## Conclusions

Acute peritonitis caused by cecal perforation can closely mimic peritoneal carcinomatosis across clinical, radiologic, and intraoperative domains. This case illustrates the diagnostic challenge posed by inflammatory conditions that produce tumor-like peritoneal nodularity and ascites. Although malignancy was strongly suspected based on systemic symptoms and imaging findings, definitive diagnosis ultimately relied on histopathologic evaluation. In the absence of malignancy or inflammatory bowel disease, the perforation was most consistent with an acute inflammatory process leading to secondary peritonitis. Recognition of such inflammatory mimics is essential to avoid diagnostic anchoring and to guide appropriate surgical and medical management.
